# Feeding Ecology of *Coryphaenoides rupestris* from the Mid-Atlantic Ridge

**DOI:** 10.1371/journal.pone.0010453

**Published:** 2010-05-03

**Authors:** Odd Aksel Bergstad, Guro Gjelsvik, Christoffer Schander, Åge S. Høines

**Affiliations:** 1 Institute of Marine Research, Arendal, Norway; 2 Norwegian Directorate of Fisheries, Bergen, Norway; 3 Department of Biology and Centre for Geobiology, University of Bergen, Bergen, Norway; 4 Institute of Marine Research, Bergen, Norway; Smithsonian's National Zoological Park, United States of America

## Abstract

The Macrourid fish roundnose grenadier, *Coryphaenoides rupestris*, is one of the most common benthopelagic fishes on the northern mid-Atlantic Ridge. The ecology of the species is comparatively well studied in continental slope waters of the North Atlantic, but not on the mid-Atlantic Ridge, which is a central mid-ocean area of its distribution. In total, 166 specimens from the RV *G.O. Sars* cruise in July 2004 were examined. The diet mainly comprised cephalopods, pelagic shrimps and fish. Pelagic and benthopelagic copepods were the most numerous prey, but did not contribute much on a weight basis. Cephalopods were by far the most important prey of the small grenadiers, while shrimps and fish became increasingly significant with increasing size. Previous studies from other areas have also found pelagic prey to be important, but in contrast to this study, cephalopods were generally of less importance. The study was an element of more wide-ranging food-web studies of the mid-Atlantic Ridge macro- and megafauna communities within the international MAR-ECO project.

## Introduction

The mid-Atlantic Ridge, a section of the global system of mid-ocean ridges, is a spectacular topographic feature of the Atlantic Ocean stretching from north of Iceland to the Southern Ocean, representing the spreading zone between the Eurasian and American continental plates. Although mid-ocean ridges have an extensive distribution and cover 22% of the Earth's surface [1], these remote areas are largely unexplored. The knowledge about the animal communities and biology and ecology of individual species in these waters remains limited. The rugged terrain and great depths make the ridges particularly challenging study areas.

To learn more about animal life on the mid-Atlantic Ridge, the international project MAR-ECO was initiated in 2001 [2]. One of the main tasks was to identify trophic relationships and model food web patterns. To facilitate such analyses, feeding ecology data for a range of vertebrates and invertebrates were collected.

The macrourid roundnose grenadier (*Coryphaenoides rupestris*) is a typical demersal fish of the North Atlantic slope waters [3], [4] and it was the most abundant species amongst the 16 macrourids recorded on the mid-Atlantic Ridge [5], [6]. This makes it a particularly interesting species for trophic studies. It is also a commercially exploited fishery resource on the mid-Atlantic Ridge (e.g. [7]). This study aims to derive information on diet composition and thereby trophic ecology of the grenadier by classical numerical and gravimetric analyses of the stomach contents.

## Materials and Methods

**Figure 1:**
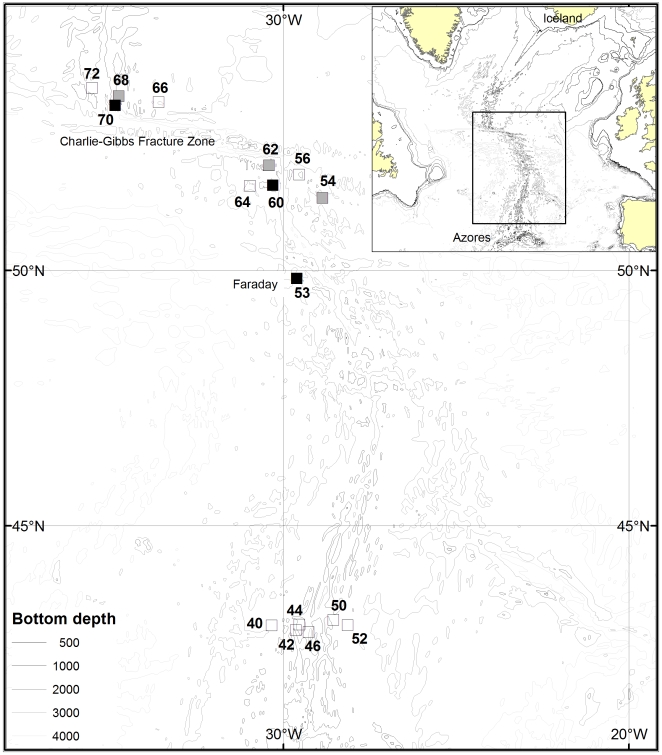
Bottom trawl sampling locations on the 2004 expedition on RV *G.O. Sars*. Filled squares: locations with catches of *C. rupestris*, among which the black squares denote locations where diet samples were collected. Open squares: locations with no catch of *C. rupestris*.

The material used in this study was collected on Leg 2 of the MAR-ECO cruise on the RV *G.O. Sars* in the period 4 July- 5 August 2004. During Leg 2 the vessel operated on the mid-Atlantic Ridge (Fig. 1) north and south of the Charlie-Gibbs Fracture Zone at about 52° N, and north of the Azores at about 42° N. Pre-planned efforts were concentrated in these two subareas, and an additional two trawls were made on the Faraday Seamount.

### Sampling

The 16 stations sampled successfully with the bottom trawl are shown in Figure 1. *C. rupestris* occurred only on the Faraday Seamount and north and south of the Charlie-Gibbs Fracture Zone and samples from three stations 53, 60 and 70 where the grenadier was most abundant were used in the diet studies (Table 1, [5]). Station 53 was sampled twice and both trawls captured roundnose grenadier. A double warp bottom trawl (Campelen 1800 shrimp trawl) fitted with ‘rockhopper’ ground gear was applied for collecting the samples (Further details in [8]).

**Table 1:** RV *G.O. Sars* 2004 MAR-ECO expedition trawl stations were grenadier were collected.

Sampling area	Faraday seamount	South of Charlie-Gibbs Fr. Zone	North of Charlie-Gibbs Fr. Zone
Station	53	60	70
Date	2004-07-15	2004-07-19	2004-07-26
Latitude (N)	49°86′	51°56′	52°98′
Longitude (W)	29°63′	30°31′	34°87′
Trawl speed (knots)	2.4	2.4	2.2
Start time of tow	19∶22	11∶15	16∶00
Duration of trawl (min)	28	19	16
Trawling distance at bottom (nautical miles, = 1852 m)	1.11	0.77	0.60
Average depth (m)	966	1255	1650
Number of *C. rupestris* examined	40	73	53
Number of stomachs with contents	34	53	28

All roundnose grenadiers were sorted out from the total catch, weighed and counted. Specimens that had everted stomachs were common and were discarded. Specimens that appeared to have intact stomach contents were frozen onboard.

For every specimen, pre-anal fin length to nearest cm below, and ungutted weight (g) were recorded. Since several specimens had broken or regenerated tails, pre-anal length was chosen over total length. The stomach contents were extracted from half-thawed specimens, and preserved on 70% ethanol.

Prey animals were identified to lowest possible taxonomic level. Advanced digestion often made identification to a lower taxonomic level impossible. If possible total body length or another conventional taxon-specific body-size measure was recorded. When fragments of recognizable prey animals were found, the minimum number of specimens the fragments could have originated from was estimated. All prey animals/categories were dried on paper to remove excess water and then weighed to the nearest 0.001 g. Animals that weighed less than 0.001 g, were assigned a weight 0.0001 g.

### Analyses

The proportion in terms of weight (% W) of each prey category was calculated as
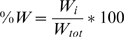
where W_i_  =  Weight of prey category i, and W_tot_  =  Weight of total stomach contents.

The proportion in terms of numbers (% N) in each prey category was calculated in a similar way:
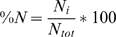
Where N_i_  =  Numbers of prey category i, and N_tot_  =  Total number of prey specimens.

The frequency of occurrence (% F) was calculated as
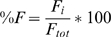
Where F_i_  =  Frequency of occurrence of prey category i, and F_tot_  =  Total number of stomachs with contents.

Of the cephalopod fraction, 17% was unidentifiable tissue. Only four specimens were intact and could be identified to species (*Gonatus fabricii*). Two of the *G. fabricii* were large and lightly digested, and contributed ¼ to the total weight of the stomach contents of all the grenadiers examined. If included in the analyses of relative composition of the contents, these two specimens would lead to a significant bias by inflating the percentage of cephalopods to a very high and unlikely level. While recognising the existence in the contents of these cephalopods, it was considered reasonable to exclude them from the weight analyses.

Cephalopod beaks were identified by Uwe Piatkowski and Nina Keul at IFM-Geomar, University of Kiel and upper and lower rostral length measured. Lower rostral length is suitable for estimating cephalopod length and weight using relationships established by Clarke (1986) [9]. In this study, the relationship for *Gonatus fabricii* was used:
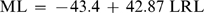


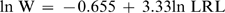
where ML  =  Mantle length, LRL  =  Lower rostral length, and W  =  Weight. Mantle length was also measured on intact cephalopod specimens.

Prosoma length was measured on copepods, while carapax length was used for shrimps, euphausiids and *Gnathophausia* zoea. In order to analyse size-related variation in diet composition, the prey composition for four predator length groups was derived. The predator groups had pre-anal fin lengths <11 cm, 11–13 cm, 14–16 cm, and >16 cm. The smallest and largest specimens in the available material were 3.3 cm and 19.7 cm respectively. Length groups were selected so as to facilitate comparison with previous studies that used the same size categories (e.g. [10]).

## Results

A total of 166 stomachs of roundnose grenadier were examined, and of these 115 had contents. Forty-one prey categories were identified. The real number of prey categories was most likely higher, as a considerable portion (10%) of the stomach contents was too digested to identify the prey.

**Figure 2:**
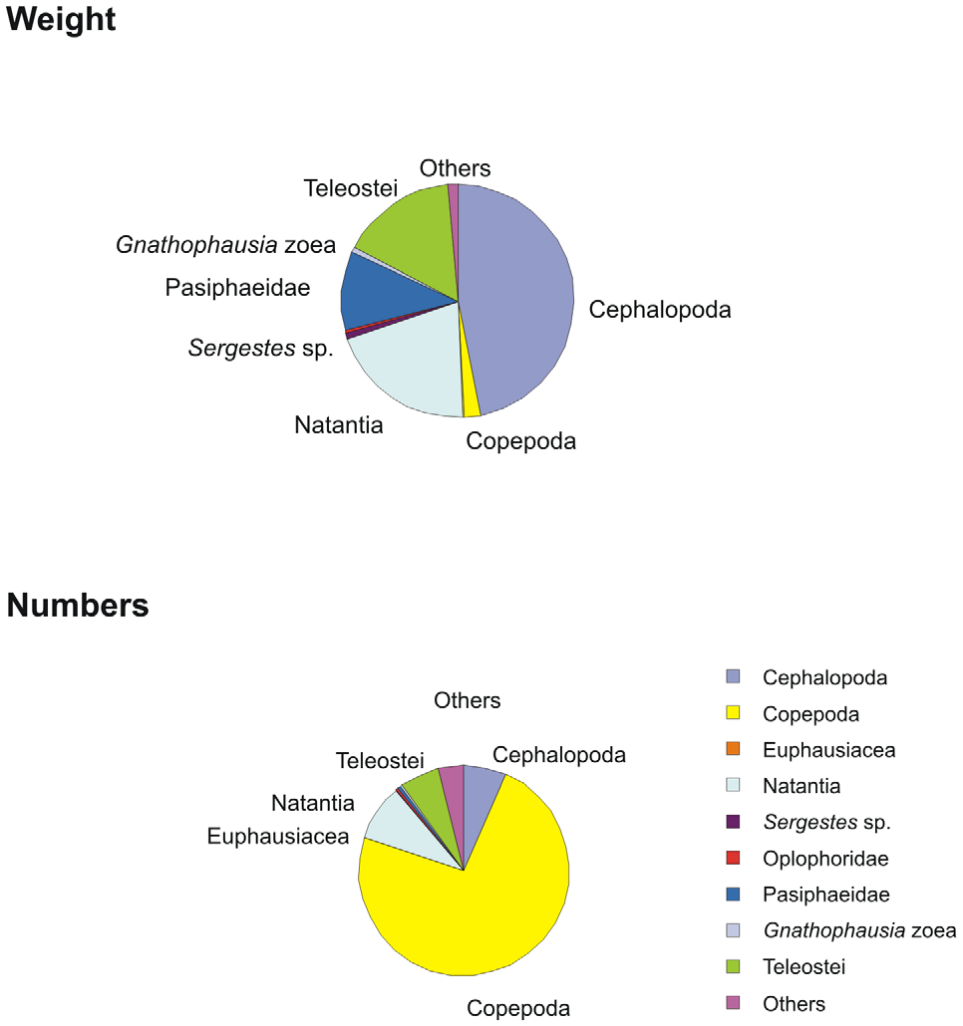
The taxonomical composition of the stomach contents of *C. rupestris* in terms of weight (upper) and numbers (lower).

Three main prey categories, cephalopods (Coleoidea), shrimps (Natantia) and fish (Teleostei), contributed 85% to the total wet weight, each with 42%, 29% and 14%, respectively (Fig. 2). Table 2 provides details on the prey composition of the pooled stomach contents, and the composition for each of four length groups of roundnose grenadier.

**Table 2:** Prey composition of roundnose grenadiers (*Coryphaenoides rupestris*).

	Predator length group, cm.											
	<11 cm			11–13 cm			14–16 cm			>16 cm		
Prey category	%W	%N	%F	%W	%N	%F	%W	%N	%F	%W	%N	%F
Hydroida							0.0	0.3	3.3			
Polychaeta, tubes							0.0	0.8	3.3			
Cephalopoda, indet.*	27.4	10.0	32.3	8.4	4.4	53.3	21.8	5.5	73.3	20.9	5.8	70.8
*Gonatus fabricii*				60.3	0.5	6.7	0.1	0.3	3.3	3.3	0.3	4.2
Crustacea, indet.	4.0	5.0	16.1	1.1	1.6	20.0	0.0	0.3	3.3	0.0	2.4	8.3
Copepoda, calanoida	2.5	36.0	54.8	0.6	39.3	70.0	0.9	36.0	76.7	0.7	31.5	83.3
*Bathycalanus richardi*							0.1	0.3	3.3			
*Calanus hyperboreus*							0.0	0.5	6.7			
Aetideidae				0.0	3.3	26.7	0.2	4.5	30.0	0.1	4.1	33.3
*Gaetanus* sp.							0.0	0.3	3.3			
*Euchirella* sp.				0.0	0.3	3.3						
*Undeuchaeta* sp.				0.0	0.3	3.3	0.0	0.5	6.7			
*Pseudochirella* sp.	0.2	1.0	3.2	0.5	11.5	33.3	1.1	15.0	50.0	0.9	18.3	62.5
*Pseudochirella pustulifera*							0.0	0.3	3.3			
Euchaetidae	0.3	1.0	3.2	0.0	0.5	6.7	0.0	0.3	3.3	0.0	0.3	4.2
*Euchaeta* sp.							0.1	1.0	13.3	0.0	0.3	4.2
*Pareuchaeta* sp.	0.2	1.0	3.2	0.0	1.9	13.3	0.1	1.5	16.7	0.0	2.4	12.5
*Pareuchaeta norvegica*				0.1	1.1	6.7	0.1	1.5	20.0	0.1	2.4	16.7
*Pseudeuchaeta* sp.							0.0	0.5	3.3			
Metridiidae	0.3	1.0	3.2				0.0	0.3	3.3			
*Metridia* sp.							0.0	0.5	6.7	0.0	0.7	8.3
*Metridia macrura*							0.0	0.3	3.3			
Lucicutiidae	0.1	3.0	6.5	0.1	13.2	33.3	0.1	6.5	36.7	0.0	3.4	20.8
Heterorhabdidae							0.0	0.3	3.3			
*Heterorhabdus* sp.	0.0	1.0	3.2	0.0	0.3	3.3	0.0	0.5	6.7	0.0	0.3	4.2
*Oncaea* sp.				0.0	0.3	3.3						
Monstrillidae										0.0	0.3	4.2
Euphausiacea										1.1	0.3	4.2
Natantia, indet.	3.7	8.0	25.8	6.4	4.9	43.3	38.9	8.3	66.7	17.9	10.8	70.8
*Sergestes* sp.				0.5	0.3	3.3				1.5	0.3	4.2
Oplophoridae							1.5	0.8	3.3			
Pasiphaeidae				7.1	0.5	3.3	10.1	0.5	6.7	14.1	0.7	4.2
*Gnatophausia,* zoea							0.7	0.3	3.3	1.8	0.3	4.2
Amphipoda				0.0	0.5	6.7	0.5	0.5	6.7			
Sipunculiodea				0.0	0.3	3.3						
Teleostei	0.1	1.0	3.2	5.5	4.1	50.0	9.9	6.0	66.7	31.8	8.1	83.3
Indet. Eggs				0.6	3.3	3.3	1.0	0.3	3.3			
Indet.	61.4	32.0	90.6	8.7	7.4	70.0	12.5	6.3	66.7	5.7	6.8	54.2
Number of stomachs examined	43			43			47			33		
Numbers everted	12			13			17			9		

*Cephalopods beaks excluded.

A total of 215 cephalopod beaks were found in the stomach contents, and 178 of these were identified to four species: *Gonatus fabricii* (153), *Taonius/Teuthowenia* sp., possibly *Taonius pavo* or *Teuthowenia megalops* (21), *Histioteuthis reversa* (3) and *Todarodes sagittatus* (1). The rest remained unidentified due to advanced digestion.

The second largest group, unidentified shrimps, constituted 18% of the total weight. The bulk was unidentifiable beyond higher taxon. Pasiphaeidae constituted 10% of the total weight, but only 1% of the total number. *Sergestes* sp. contributes 1%. Unidentified shrimps occurred in about half of the stomachs.

Teleost fish were also found in nearly half of the stomachs, but only as digested remains such as eyes, vertebrae and scales, and in a few cases muscle tissue. No otoliths were found.

Copepods were by far the most numerous prey and also had the highest frequency of occurrence (70%). But they accounted for only 2% of the total weight. The most frequent families were Aetideidae, especially the genus *Pseudochirella* sp., Euchaetidae and Lucicutiidae.

Remains of scales that may have belonged to the polychaete group Aphroditiformia had a rather high frequency of occurrence, roughly 30%. There could be as many as 30 scales in one stomach, but more commonly only a few. In terms of weight scales were insignificant.

Setae from chaetognaths were also found in 34% of the stomachs with contents. They were insignificant considered on a weight basis and probably nutritionally insignificant, but the high frequency of occurrence suggested that chaetognaths were rather common. Various other indigestible objects were also found in some of the stomachs, e.g. scales from the roundnose grenadier itself. Rocks were found in 9 stomachs, which, along with the polychaete scales, suggest occasional benthic feeding. No other sediments were found. Parasites such as nematodes and cestodes were present in a few stomachs, but identifying and quantifying these was considered outside the scope of the study.

**Figure 3:**
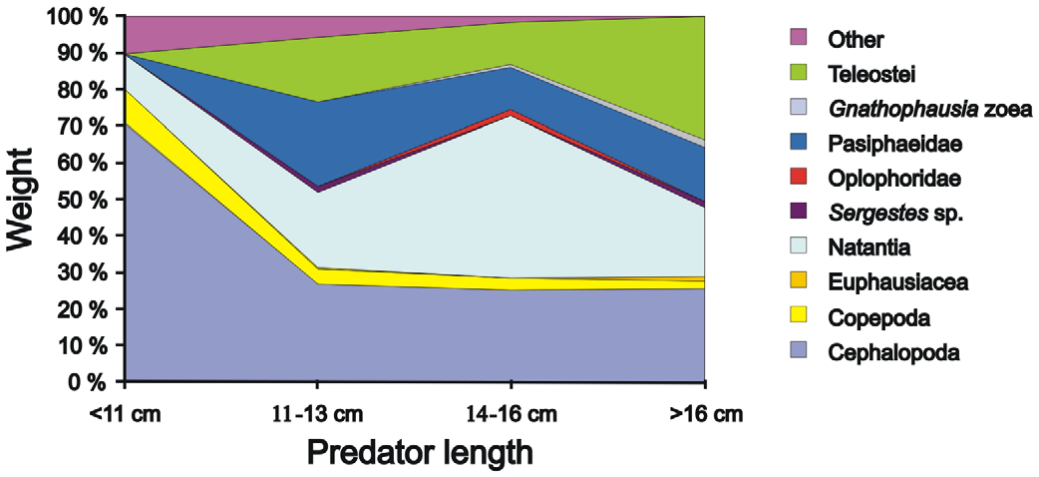
Composition of stomach contents in terms of weight of different length groups of *C. rupestris*.

The proportion of unidentifiable remains varied between predator length groups. Therefore, the unidentified remains were re-distributed proportionally on the identified prey groups. Figure 3 shows the prey composition of the different predator length groups in terms of weight. Numerical data and %F scores are given in Table 2. In this figure two cephalopods, *Gonatus fabricii*, were removed because of their very large contribution to the stomach content weight. Together they constituted more than 60% of the total weight in this predator length group (11–13 cm), and this obviously distorted the result.

Overall, the diversity of prey increased with the size of the grenadier. There were also size-related trends for individual major prey taxa. Cephalopods were important in terms of weight for all length groups, but especially for the smallest length group. The proportion of cephalopod tissue decreased with increasing predator length, from 72% in the smallest fish to around 30% in the three largest length groups (Fig. 3). This result is in some contrast with the observation that only 4% of the cephalopod beaks were found in the smallest length group, in comparison to 19, 43 and 33% in the other groups.

The second largest predator length group, 14–16 cm, had the highest portion of shrimps. But shrimps were also prominent in the other groups, and especially Pasiphaeidae was present with a considerable percentage weight in the three largest length groups. Fish (Teleostei) was another prey category of importance in the three largest length groups. *Sergestes* sp., Oplophoridae, Euphausiacea and *Gnathophausia* zoea were minor prey and no conclusions can be drawn on variation with predator size.

Copepods were evenly distributed among the length groups both in terms of weight and number. Chaetognaths were mostly found in the two smallest predator length groups, the amounts of setae decreased with increasing fish size.

For all length groups, the prey category “other” contained hydroids, polychaete tubes, amphipods and unidentified eggs. These were pooled because of their low contribution both with respect to weight and numbers.

**Figure 4:**
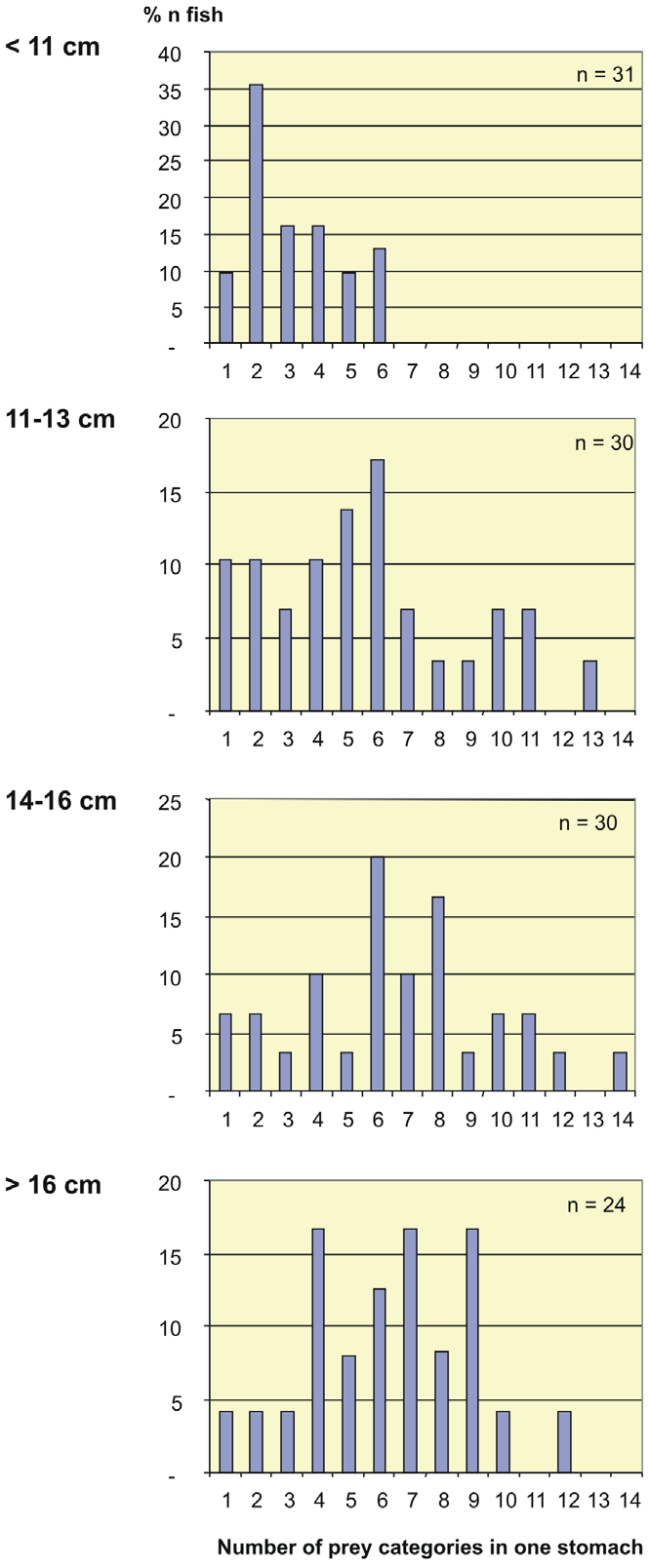
The number of prey categories per stomach for different size classes of *C. rupestris.*

The number of different prey categories per stomach ranged from 1 to 14 (Fig. 4). Mean number of prey categories was 5.4, and the smallest predator length group had the lowest number of prey categories. The prey animals were identified to varying taxonomic levels, and therefore the results do not truly reflect prey species diversity. Over half of the prey categories were copepods, and in the larger grenadiers, it was quite common to find more than one copepod taxon in the same stomach.

**Figure 5:**
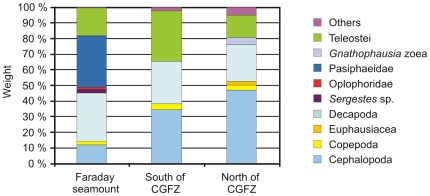
Prey composition at three different locations on the mid-Atlantic Ridge.

Samples from three different locations were examined; the Faraday seamount (superstation 53), and two locations south (superstation 60) and north (superstation 70) of the Charlie-Gibbs Fracture Zone. Figure 5 shows percentage weight of prey categories in these different locations.

Cephalopods were rather important in all three locations, but there was a large difference between the most distant locations, i.e. the Faraday seamount and the north of the Charlie-Gibbs Fracture Zone. The two heavy specimens of *G. fabricii* found in stomachs north of the Charlie-Gibbs Fracture Zone were omitted in Figure 5, but still cephalopods constituted nearly half of the total stomach contents in this area. On the Faraday seamount on the other hand, cephalopods constituted only 12%.

The shrimp fraction appeared similar in the different locations, yet Faraday seamount had a slightly higher proportion than the other stations, and it was only in that that identifiable shrimps were found. Oplophoridae and *Sergestes* sp. played a minor part, while Pasiphaeidae were prominent. Other crustaceans such as euphausids and mysids occurred only on the northernmost station, while copepods were evenly distributed in all three areas.

Fish were important in all areas, but the proportion of fish was highest at the location just south of the Charlie-Gibbs Fracture Zone.

Setae from chaetognaths occurred most frequently in the two northernmost locations, with 38 and 54% south and north of the Charlie-Gibbs Fracture Zone respectively, while on Faraday seamount they occurred in only 8% of the stomachs. Polychaete remains were more evenly distributed among the three locations where the frequency of occurrence was 24 (Faraday seamount), 45 and 31% (south and north of Charlie-Gibbs Fracture Zone), respectively.

Four specimens of cephalopods were sufficiently intact to be measurable; and they measured 5, 12, 53 and 85 mm mantle length. Many beaks could be measured, however, and using conversion equations, mantle lengths and body weights could be estimated. Based on lower rostrum length, the mantle lengths and weight of 34 specimens of *Gonatus fabricii* were calculated. The calculated mantle lengths ranged from 24 to 183 mm; with corresponding weights 2 and 132 g. Mean mantle length was 102.9 mm and mean weight 38.6 g.

**Figure 6:**
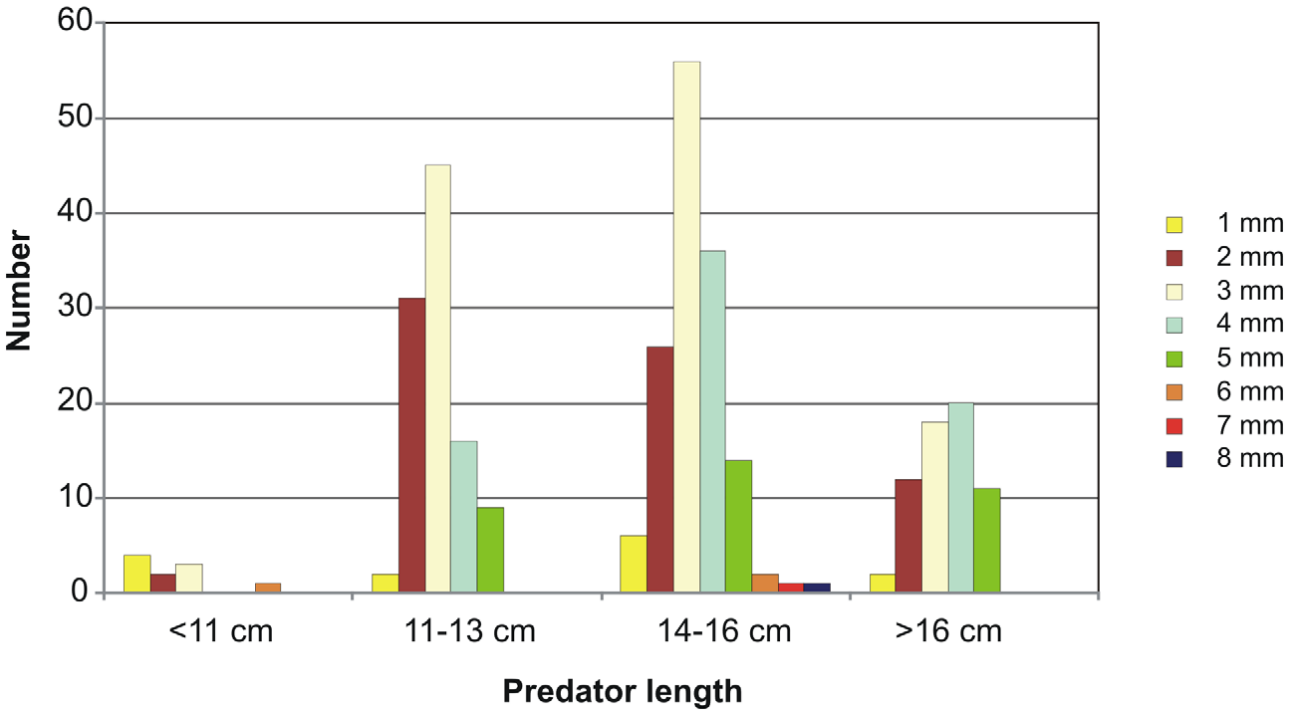
Length compositions of copepods found in stomach contents of the four predator length groups.

Copepods were abundant in the stomach contents, and 316 specimens, or about 40% of the total number was measurable. The copepods range in size from 1 mm to 8 mm, but 3 mm long (prosoma length) copepods were encountered most frequently. A shift towards slightly larger copepods was observed for the three largest length groups of roundnose grenadier (Fig. 6). Unidentified decapods shrimps were mostly too digested or fragmented to be measured, but measurable specimens ranged in size from 2 mm to 5 mm, with one exceptional specimen of 16 mm (carapax length). The one measurable specimen of *Sergestes* sp. was 25 mm long. Three specimens of shrimps from the family Oplophoridae measured 4 mm, 4 mm, and 7 mm. Only one specimen of Euphausiacea was measurable and this was 11 mm carapax length. The *Gnathophausia* zoea specimens were quite different in size, 12 and 28 mm. Other prey groups could not be measured, including all the fish.

## Discussion

Common concerns with regards to stomach content analyses also apply to this study. One concern is that the number of predator specimens suited for diet studies is often much lower than the total number sampled. The number of specimens available for analyses in this study was limited by two factors. Due to time constraints only 17 bottom trawl tows were made, and not all of these were at depths where roundnose grenadier occurred. Also, when the species was abundant, many specimens had evacuated the stomachs and were useless for diet analyses. About 1700 specimens of roundnose grenadier were caught on the 2004 MAR-ECO cruise, but ¾ were discarded at sea after having been counted, measured and weighed. Only the specimens with apparently intact stomachs were frozen for further examination. Slower hauling speed may reduce the stomach eversion problem, but since the macrourids have gas-filled swim bladders, the problem tends to be difficult to overcome.

Diet analyses may for various reasons not reveal unbiased information on the diet composition. Advanced digestion made a considerable portion of the stomach contents unidentifiable. Digestion rates are different for different prey categories, and prey with hard parts and exoskeletons tend to become overrepresented compared to soft-tissued, easily digested prey. In the roundnose grenadier stomachs remains of chaetognaths and probably aphroditiform polychaetes were found, but since there were no tissue, these prey categories were insignificant in terms of weight, and their relative importance was difficult to determine.

Remains from cephalopods and fish were often difficult to distinguish, and as a rule of thumb, if no vertebrae were found, these remains were reported as cephalopod. This may have led to some overrepresentation of cephalopods. Cephalopod remains could also be difficult to distinguish from unidentifiable soft tissue.

An alternative strategy to that chosen in this study is to “reconstruct” the prey composition using length-weight conversions for prey actually observed in the stomach contents. This is a reasonable approach when the majority of the contents can be counted and measured, but not very useful when a large proportion of the contents is unidentifiable and/or immeasurable, as in this study.

In principle, reconstruction can be applied to individual prey groups, e.g. cephalopods. In this study only the lengths of the cephalopods was studied based on beak sizes, but it is also possible to derive original body weights and thus diet contribution in terms of weight. In the case of *Gonatus fabricii*, 34 lower beaks and 119 upper beaks were identified. The total estimated biomass from the lower beaks of *G. fabricii* was 1114 g. This is roughly 10 times the weight of the total prey animals found in all the 115 grenadiers, and still there are 119 upper beaks for which no regressions were available. Cephalopod beaks are thought to accumulate in stomachs, and this seemed to be the case in this study. E.g. in one stomach, six lower *G. fabricii* beaks were found, and together they had an estimated weight of 211.9 g, which is nearly the double of the weight of the total stomach contents. High numbers of beaks should not alone be interpreted as reflecting very high consumption rates. It is not known for how long cephalopod beaks are retained in stomachs. The different numbers of upper and lower beaks may be due to different digestion rates or different evacuation rates.

An apparent discrepancy occurred in the smallest length group that had the highest proportion of cephalopod remains, but the least occurrence of cephalopod beaks. The small grenadiers may feed on juvenile cephalopods; if the beaks are small they may pass through the digestive system. Or maybe the smaller fish digest the beaks faster, not to clog the digestive system. Disregarding length groups, 45% of the cephalopod remains did not contain any beaks, which may mean that the grenadier has taken a bite of a part of the cephalopod rather than eating the whole animal.

As in many other studies unidentifiable remains were found quite frequently. Obtaining DNA from stomach contents is difficult but possible [11], [12]. However, identification using molecular methods was considered outside the scope of this study. Future DNA-based stomach content analyses would greatly be aided by DNA-libraries specially focussing on DNA-identification (i.e. DNA barcoding) [13].

The sources of errors and imprecision discussed above are potentially serious and the results should therefore be treated with caution, as should all other similar investigations using the same approach. The main unknown is what prey species were missed or underestimated due to advanced or rapid digestion. Despite the shortcomings, the study reveals some major patterns that provide new and useful information on the trophic ecology of the grenadier.

The diet of roundnose grenadier from the mid-Atlantic Ridge was quite diverse, and can be characterised as pelagic with possible benthic elements. The most important prey were cephalopods, shrimps and fish, supplemented with a variety of other small organisms, with a notable proportion of pelagic copepods. The cephalopods and shrimps identified are mainly pelagic, but what appeared to be benthic polychaete remains were also found. Copepods were the most numerous prey animals, however on a weight basis the four specimens of *Gonatus fabricii* made the largest contribution. According to the beaks found in the stomach, *G. fabricii* was by far the most frequently eaten cephalopod, but also *Taonius/Teuthowenia* sp. was common. *Gonatus* sp. was the most abundant taxon of cephalopods captured by midwater trawls on Leg 1 of the MAR-ECO cruise [14], and this suggests that the extensive consumption of *Gonatus* is due to high availability. One of the most striking features was the change in diet as the roundnose grenadiers became larger; the cephalopod proportion was decreasing with increasing predator size, while the proportion of fish and shrimps increased.

The samples used in this study were from three different locations (north and south of the Charlie-Gibbs Fracture Zone, and the Faraday seamount) and this was reflected in the diet; the shrimp proportion was roughly equal, the cephalopod proportion ranged from 12% to 47%, and the fish proportion ranged from 14% to 32%. Pasiphaeidae, Oplophoridae and *Sergestes* sp. only occurred on the Faraday seamount. Euphausids only occurred on the northernmost station, while mysids were found in small amounts on Faraday seamount (1%) and on the northernmost location (4%). The greater variance in prey on the Faraday seamount is certainly interesting, but the number and frequency of occurrence is rather low, so this result can be incidental. It is therefore difficult to draw a firm conclusion, but the diet on the Faraday seamount does appear slightly more diverse.

The dominance of pelagic prey in the diet indicates that the roundnose grenadier moves off the bottom to forage, a conclusion also drawn by others [15]–[17]. This is supported by several reports of pelagic occurrence of roundnose grenadier; [18], [15], [19], [20]. Only 11 specimens of roundnose grenadier were captured during pelagic trawling on the MAR-ECO cruise (Leg 1), but these were juveniles and had a total weight of 72 g. Mauchline and Gordon (1991) noted that much was yet unknown about the frequency and duration of the pelagic excursions and suggested for the Rockall Trough, based on the few pelagic observations, that “such excursions are rare or on a short-time scale [21]. Also, plankton and pelagic nekton may perform vertical migrations, and may thus be found close to the bottom during the day [21], [22]. This, in addition to pelagic foraging, may explain the high proportion of pelagic prey in the roundnose grenadier stomach contents.

There are some similarities with previous diet studies of roundnose grenadier, but also some interesting differences, in particular regarding ontogenetic changes. Mauchline and Gordon (1984) examined roundnose grenadier from the Rockall Trough (northwest of Scotland and Ireland) [23], Bergstad et al. (2003) from the Skagerrak [10], and Jørgensen (1996) from West Greenland waters [17]. Podrazhanskaya (1971) found the diet of roundnose grenadiers in the Northwest Atlantic and Icelandic waters to be composed of *Themisto* spp. (Amphipoda), copepods, euphausids, squid, and fish [24]. Benthic prey like polychaetes and some shrimps associated with the bottom (*Pandalus borealis* and the genus *Pantophieus*) also occurred, and together with observed sand, mud and stones, the conclusion was drawn that roundnose grenadier may forage rather extensively on bottom dwelling species. This contrasts with other studies, and also with the present results. Benthic prey was not common on the mid-Atlantic Ridge where the pelagic prey was dominant.

The most striking difference between the diet on the mid-Atlantic Ridge and the other areas in the Atlantic slope waters was the greater importance of cephalopods, especially to grenadiers below 11 cm PAL. There were no cephalopods in the diet in Skagerrak, and few in the small fish from the Rockall Trough in the length groups corresponding to fish below 11 cm. Contrary to the mid-Atlantic Ridge, cephalopods were barely present in the smallest length group in West Greenland waters, but increased significantly with increasing fish size.

Crustaceans are important prey in all areas, especially shrimps, copepods and amphipods, but also euphausids and mysids were significant in some areas. In the Skagerrak in particular there was a clear dominance of crustaceans, but also in West Greenland waters during winter were the crustaceans most prominent. While euphausids were the most important prey in the smallest length group in Skagerrak, they were absent from this length group on the mid-Atlantic Ridge. Copepods were by far the most frequently encountered prey category, but were not very prominent considered on a weight basis on the mid-Atlantic Ridge, although they were important in Rockall Trough and Icelandic waters, and for fish below 11 cm in the Skagerrak. Amphipods were insignificant in the diets on the mid-Atlantic Ridge, and only four specimens were found altogether, while this was a dominating prey in Icelandic waters.

Fish were a relatively important prey in all areas, except in the Skagerrak, where few fish remains were found. Fish were found in increasing amounts with increasing predator size in both the Rockall Trough, West Greenland waters and on the mid-Atlantic Ridge.

The locations in this and previous studies were widely dispersed over a large area in the North Atlantic, but the dominating prey animals were essentially the same; crustaceans, cephalopods and fish. Different crustaceans, like shrimps, copepods, amphipods, euphausids and mysids, varied in relative importance. Locally, the diet on the mid-Atlantic Ridge was also composed of the same prey animals with different relative importance, except for some genera of shrimps which occurred only in one location. The differences on a larger scale were therefore expected and were most likely due to different availability rather than differences in preferences.

Some of the variation may be explained by seasonal variation in both the availability of prey and by migrations by the predator itself. As mentioned previously Jørgensen (1996) found seasonal variance in the diet from samples caught in the winter and summertime [17]. According to Troyanovsky and Lisovsky (1995) seasonal migrations are typical of the roundnose grenadiers in the Northwest Atlantic; they stay in the deep-waters during winter, and move up to the upper part of slope in spring, and these migrations follow the zooplankton migrations [7]. As a contrast, there are no obvious patterns of seasonal migrations on the mid-Atlantic Ridge or in the Northeast Atlantic [7]. Mauchline and Gordon (1984) sampled throughout a year in the Rockall Trough, but no seasonal changes were found [23].

Interesting next steps would be to compare feeding patterns among the co-occurring 16 or more macrourid fishes inhabiting the mid-Atlantic Ridge [6], and to explore the ecological roles played by macrourids in the demersal food web of the ridge system.
